# Case reports of juvenile GM1 gangliosidosisis type II caused by mutation in *GLB1* gene

**DOI:** 10.1186/s12881-017-0417-4

**Published:** 2017-07-17

**Authors:** Parvaneh Karimzadeh, Samaneh Naderi, Farzaneh Modarresi, Hassan Dastsooz, Hamid Nemati, Tayebeh Farokhashtiani, Bibi Shahin Shamsian, Soroor Inaloo, Mohammad Ali Faghihi

**Affiliations:** 1 Pediatric Neurology Department, Pediatric Neurology Research Center, Shahid Beheshti University of Medical Sciences (SBMU), Tehran, Iran; 2 Diagnostic Laboratory Sciences and Technology Research Center, School of Paramedical Sciences, Shiraz University of Medical Sciences, Shiraz, Iran; 3 Center for Therapeutic Innovation, Department of Psychiatry and Behavioral Sciences, University of Miami Miller School of Medicine, Miami, USA; 4 Comprehensive Medical Genetic Center, Shiraz University of Medical Sciences, Shiraz, Iran; 5 Pediatric Congenital Hematologic Disorders Research Center, Shahid Beheshti University of Medical Sciences, Tehran, Iran; 6 Neonatal Research Center, Shiraz University of Medical Sciences, Shiraz, Iran; 7 Shiraz Neuroscience Research Center, Shiraz University of Medical Sciences, Shiraz, Iran

**Keywords:** GM1 gangliosidosis, *GLB1*, Case report

## Abstract

**Background:**

Type II or juvenile GM1-gangliosidosis is an autosomal recessive lysosomal storage disorder, which is clinically distinct from infantile form of the disease by the lack of characteristic cherry-red spot and hepatosplenomegaly. The disease is characterized by slowly progressive neurodegeneration and mild skeletal changes. Due to the later age of onset and uncharacteristic presentation, diagnosis is frequently puzzled with other ataxic and purely neurological disorders. Up to now, 3–4 types of GM1-gangliosidosis have been reported and among them type I is the most common phenotype with the age of onset around 6 months. Various forms of GM1-gangliosidosis are caused by *GLB1* gene mutations but severity of the disease and age of onset are directly related to the position and the nature of deleterious mutations. However, due to its unique genetic cause and overlapping clinical features, some researchers believe that GM1 gangliosidosis represents an overlapped disease spectrum instead of four distinct types.

**Case presentation:**

Here, we report a less frequent type of autosomal recessive GM1 gangliosidosis with perplexing clinical presentation in three families in the southwest part of Iran, who are unrelated but all from “Lurs” ethnic background. To identify disease-causing mutations, Whole Exome Sequencing (WES) utilizing next generation sequencing was performed. Four patients from three families were investigated with the age of onset around 3 years old. Clinical presentations were ataxia, gate disturbances and dystonia leading to wheelchair-dependent disability, regression of intellectual abilities, and general developmental regression. They all were born in consanguineous families with no previous documented similar disease in their parents. A homozygote missense mutation in *GLB1* gene (c. 601 G > A, p.R201C) was found in all patients. Using Sanger sequencing this identified mutation was confirmed in the proband, their parents, grandparents, and extended family members, confirming its autosomal recessive pattern of inheritance.

**Conclusions:**

Our study identified a rare pathogenic missense mutation in *GLB1* gene in patients with complex neurodevelopmental findings, which can extend the list of differential diagnoses for childhood ataxia in Iranian patients.

## Background

GM1 Gangliosidosis, which is a lysosomal storage disease (LSD) [1] is one of the rare neurometabolic disorders characterized by the deficiency of the enzymatic activity of ganglioside- beta-Galactosidase [2]. This deficiency causes the accumulation of glycolipids, keratan sulfate, and GM1 gangliosides in several tissues, particularly, in neurons of peripheral and central nervous system [3]. Clinical presentation of the disease reflects the neurodegenerative processes in the brain and spinal cord due to the accumulation of ganglioside-β-Galactosidase substrate. Several studies in animal models have shown that the accumulation of GM1 gangliosides in local microglial cells of central nervous system can lead to the increased activation and infiltration of inflammatory cells in these cells. The inflammation seems to play an important role in pathogenesis of the disease and its neurological manifestations [4]. GM1 gangliosidosis is estimated to occur in 1 per 100,000 to 200,000 newborns [5]. Until now, different types of GM1-gangliosidosis have been described. The most severe and frequent form of the disease is GM1 gangliosidosis type I, which is typically presented in early infants with hepatosplenomegaly, bone alteration as dysostosis multiplex, coarse faces, macrocephaly and growth retardation [6]. Patients with GM1 gangliosidosis Type I usually die before age of 3 years due to repeated bronchopneumonia [7, 8]. It is worth noting that cherry red spot is observed in more than 50% of patients with this type. In type II of the disease which is considered as juvenile or late infantile GM1 gangliosidosis, the onset of clinical presentations is delayed with slow progression. In this type, the first clinical manifestation is ataxia followed by dystonia and spasticity. In type II of the disease, patients usually do not present distinctive facial features, hepatosplenomegaly and cherry red spot, making the diagnosis perplexed. These patients have normal development at early stage of life, but they begin to develop signs and symptoms of the condition under 3 years of age in the late infantile and between 3 and 5 years in juvenile form of the disorder. Regardless of the age of onset, individuals with GM1 gangliosidosis type II usually show progressive regression of neurodevelopmental milestones, which result in the loss of motor and language skills and they might have refractory seizure [9]. GM1 gangliosidosis type III, which is considered as the adult or chronic form manifests the mildest end of the disease spectrum. The onset age of symptoms varies in this type, but it mainly represents in teens. Its characteristic features include dystonia and the spinal bones abnormalities. Life expectancy varies among people with GM1 gangliosidosis type III and most individuals with type III are of Japanese descent [10–12]. With the limited available therapeutic options, patients with GM1 gangliosidosis deteriorate progressively, leading to wheelchair-dependent disability and eventually to vegetative state and death during their 3rd decade of life.

The ganglioside-beta-galactosidase gene (*GLB1*) is located on the short arm of the chromosome 3 (3p21.33) and its encoded protein is responsible for breakdown of GM1 ganglioside in the nerve cells. Mutations in this gene are the only genetic causes of various forms of GM1 gangliosidosis. More than 165 disease-causing mutations have been identified in *GLB1* gene, mainly missense and nonsense mutations, as well as insertions and deletions with different size [13]. Partial or complete absence of *GLB1* gene product leads to the elimination of beta-galactosidase activity and accumulation of GM1 ganglioside to toxic level in many tissues and organs, mainly in the brain [14]. Progressive damage caused by the buildup of toxic substrate results in the destruction of neurons in the brain, gradual neurodegeneration and appearance of signs and symptoms of GM1 gangliosidosis [15]. In general, severity of the disease depends on residual activity of the beta-galactosidase enzyme, which in turns depend on the site and nature of the mutated nucleotide. Individuals with higher beta-galactosidase activity usually have milder signs and symptoms and later age of onset [16]. Therefore, *GLB1* mutation analysis must be considered in the infants with lysosomal storage disease as well as in the patients with ataxia and any other childhood and juvenile neurodegenerative symptoms [17].

Current study is the first report of GM1 gangliosidosis type II in Iranian population. We investigated three consanguineous families, including four patients, and all patients showed autosomal recessive form of GM1 gangliosidosis type II.

## Case presentation

Here we report four cases affected by GM1 gangliosidosis type II from three consanguineous families.

### Family I, Patient I

**Fig. 1:**
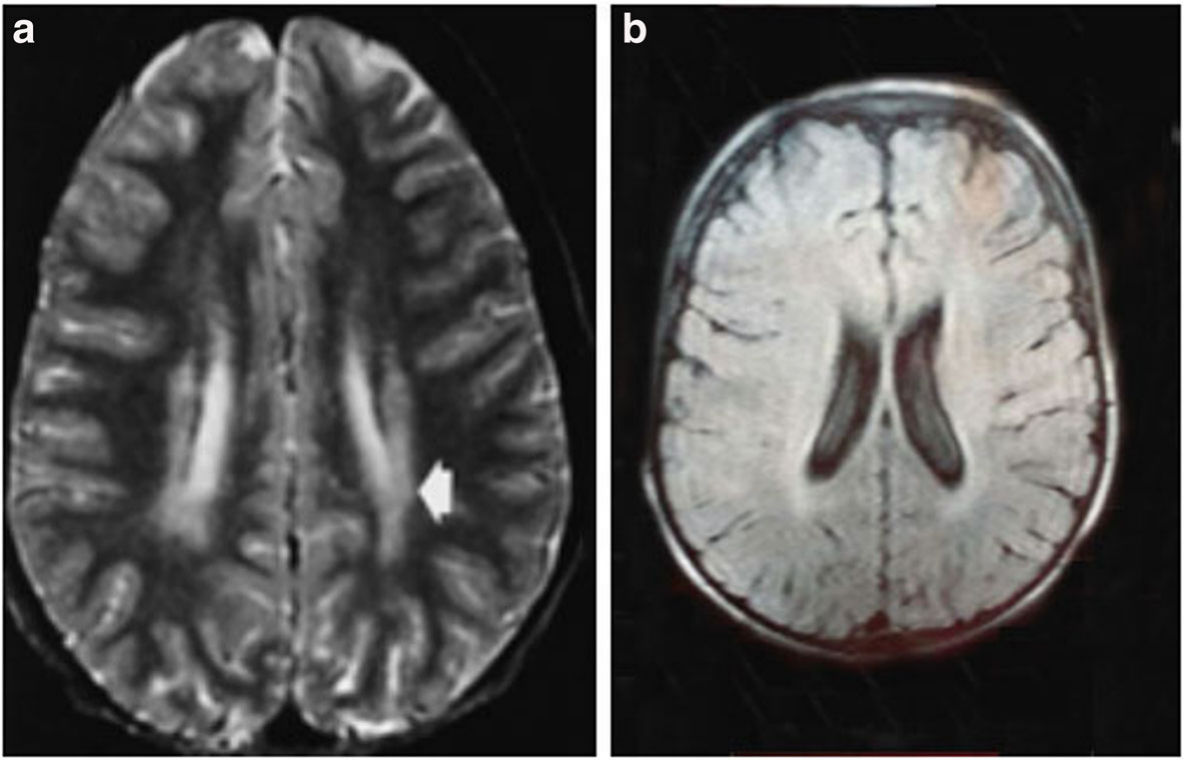
MRI report of patient I. **a** mild abnormal signal changes in periventricular white matter, **b** mild abnormal signal changes in periventricular white matter in T1 sequence

A 6.5-year-old girl was referred to the Pediatric Neurology Department in the Mofid Children Hospital, Tehran, Iran due to progressive ataxia and neurodevelopmental regression. She was born from a consanguineous marriage (first-degree cousins) with uneventful birth history. She had normal neurodevelopmental milestones up to the age of three years when she developed progressive ataxia. She gradually lost her lingual and motor skills and became wheelchair-dependent by the age of 5 years. Although she was reported to have normal intellectual abilities, her mental abilities decreased over the course of disease. Detailed neurological exam was performed in the first admission and in the subsequent visits, which revealed neurodevelopmental regression, reduction of age-related intellectual abilities and nystagmus. The “Fix and Follow test” of moving objects was not normal and she gradually lost her vision. Two magnetic resonance imaging (MRI) (in the third and fourth) assessments revealed minor signal changes in the periventricular white matter without any progression (Fig. 1a and b). Ophthalmologic study did not show any pathologic signs and all metabolic studies were reported in normal range. Electroencephalography (EEG), Visual Evoked Potential (VEP) and Electroretinography (ERG) were normal. The leukocyte enzyme activity was tested and reported normal beta-Galactosidase activity. Regarding her family history, 4 members in her mother’s family died due to similar clinical presentations.

### Family II, Patient II & III

**Fig. 2:**
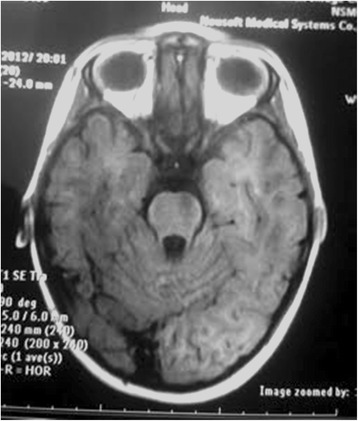
MRI image of Patient II. It indicates mild cerebellar atrophy in an axial Image of brain MRI

The family had two affected individuals who were referred to the Pediatric Neurology Department in the Mofid Children Hospital, Tehran, Iran due to neurodevelopmental regression. These two siblings, a 9-year-old boy and a 4-year-old girl, had normal developmental milestones up to the age of 3 years. The boy showed progressive ataxia, dystonia and mental decline. All metabolic studies and routine laboratory data were in normal ranges. MRI study showed mild cerebellar atrophy with mild signal changes in the periventricular white matter (Fig. 2). The affected girl had similar symptoms with loss of her acquired motor and lingual abilities after the age of 3 years as well as progressive ataxia, speech problem and neurodevelopmental regression. The parents are first-degree cousins who have one unaffected child and positive history of multiple deceased individuals with similar clinical presentation in their extended family.

### Family III, Patient IV

The patient is an 8-years old girl, who is already bed-ridden and living in the same village as the family II. She was reported to have similar findings to the family II which include progressive ataxia, neurodevelopmental regression, loss of speech and mental decline started around the age of 3 years. Her parents are first-degree cousins and have history of unexplained death of children in their extended families from both sides.

**Fig. 3:**
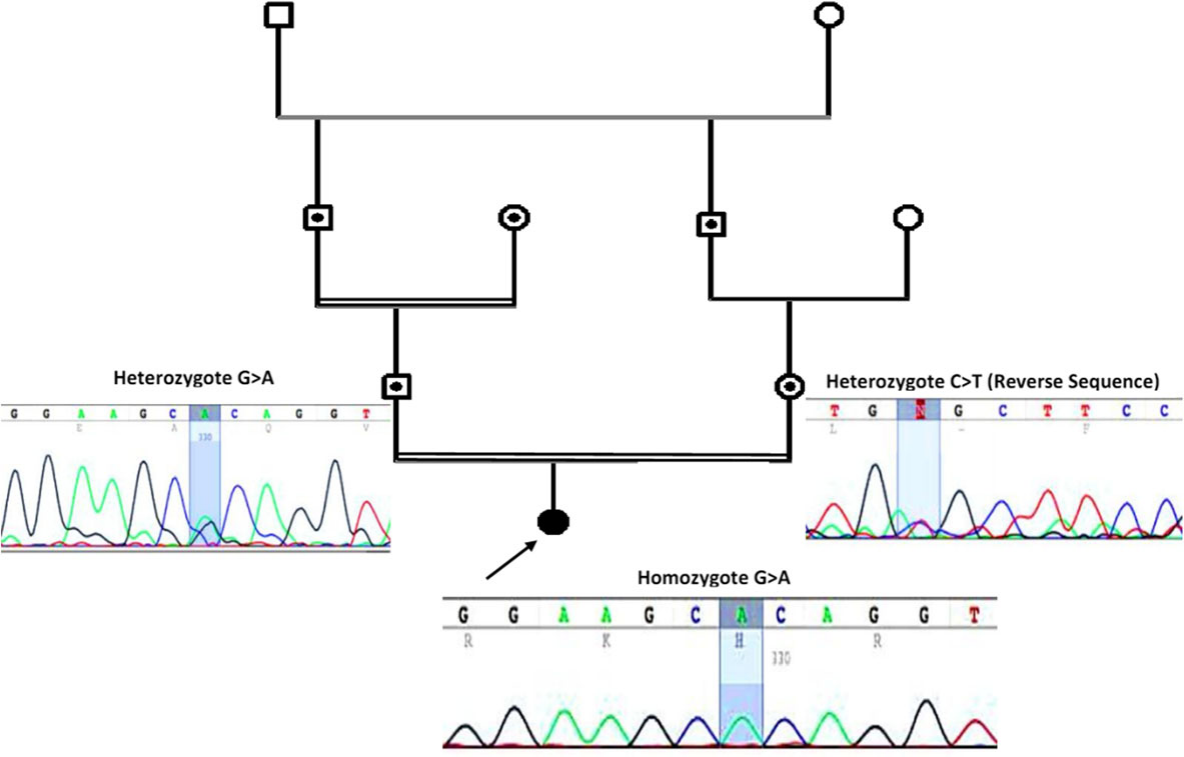
Pedigree and sanger sequencing details of Family I. Using Sanger sequencing, the inheritance mode of autosomal recessive was confirmed in this family based on identified heterozygote mutation in parents and homozygote in the proband. Sanger sequencing was performed on other patients, parents and their extended family members (data not shown)

Whole exome sequencing utilizing next generation sequencing on an Illumina platform was carried out on DNA samples from two patients and a carrier mother. Detail of next generation sequencing data are provided in Table 1. The text files of sequences were aligned using BWA aligner tool and variants were identified using GATK and annotated with the use of annovar software. In total, more than 120 K annotated variations were identified with hetero/homo ratio of 1.6 to 1.8, which then were filtered based on their frequency, location, functional consequences, inheritance pattern and more importantly clinical phenotype. All three patients were identified as homozygous for a missense mutation in *GLB1* gene (c. 601G > A, p.R201C, NM_000404). This single nucleotide variant (SNV) is located on exon 6 of *GLB1* gene in position 33099713 on chromosome 3. Homozygous mutations in *GLB1* gene have been previously reported to be the genetic cause of GM1 gangliosidosis, (OMIM number 203600), which has an autosomal recessive form of inheritance. This mutation (rs72555360) has the minor allele frequency (MAF) of less than 0.0002 and is predicted to be damaging and highly conserved using different bioinformatics software such as polyphen, SIFT, LRT, Mutation Taster, FATHMM, Radial SVM and Mutation Assessor (Table 2). The mutation was reported to cause GM1 gangliosidosis type II and clinvar ID of RCV000000973.1 for its pathogenic mutation was previously assigned. The Arg201Cys mutation, eliminating a BspMI site, was previously identified in a late-infantile/juvenile patient who had psychomotor retardation/deterioration, dysostosis multiplex, and hepatosplenomegaly. In their study, they identified that the expression of enzyme activity was less severely affected in Arg201Cys mutant which was reported to be associated with late-infantile/juvenile forms [18]. In our study, in addition to the confirmation of mutation with the use of different bioinformatics software, segregation studies using sanger sequencing was performed in family members and extended family members of the patients which confirmed the damaging possibility of the mutation in homozygote state in these patients (Fig. 3 shows one example of sequencing results and pedigree of patient 1 and their family members).

**Table 1:** Whole Exome Sequencing detail of coverage and number of reads for patient one family I. Other whole exome sequencing results have similar depth and coverage metrics

Type	Value	Type	Value
Number of mapped reads	41,674,840	Percent reads on target	95.70%
Number of amplicons	293,903	Total assigned amplicon reads	39,882,524
Percent assigned amplicon reads	95.70%	Average reads per amplicon	136
Uniformity of amplicon coverage	86.30%	Amplicons with at least 100 reads	53.69%
Amplicons with at least 1 read	99.54%	Amplicons with at least 500 reads	0.70%
Amplicons with at least 20 reads	90.02%	Amplicons reading end-to-end	35.97%
Amplicons with no strand bias	85.64%	Total aligned base reads	7,342,243,527
Bases in target regions	57,742,646	Total base reads on target	6,979,820,754
Percent base reads on target	0.95	Uniformity of base coverage	0.85
Average base coverage depth	121	Target bases with no strand bias	78.31%
Target base coverage at 1x	99.18%	Target base coverage at 100x	47.95%
Target base coverage at 20x	87.91%	Target base coverage at 500x	0.62%
Percent end-to-end reads	58.98%	mapping rate	99.10%
AQ17	92.21%	AQ20	87.51%

**Table 2:** Identified mutation in *GLB1* gene in the probands and the consequences of mutation using different bioinformatics analysis

Chr	Position	Ref	Alt	Location	Gene	SNP_ID	Function
3	33099713	G	A	exonic	GLB1	rs72555360	nonsynonymous
Transcript_ID	Exon	cDNA	Protein	Transcript_ID	Exon	cDNA	Protein
NM_000404	exon6	c.C601T	p.R201C	NM_001079811	exon6	c.C511T	p.R171C
ESP_Frequency	1000g_freq	SIFT	Prediction	Polyphen	Prediction	LRT_score	LRT_pred
0.0002	.	0.01	Damaging	1	Damaging	0	Damaging
MutationTaster	Prediction	MutationAssessor	Prediction	FATHMM	Prediction	RadialSVM	Prediction
1	A	3.435	Medium	−4.76	Damaging	1.085	Damaging
SNP_ID	Clinvar_ID	Clinvar_ID	Disease	LR_score	LR_pred	VEST3_score	CADD_raw
rs72555360	pathogenic	RCV000000973.1	Juvenile_GM > 1 < _gangliosidosis	0.974	Damaging	0.947	4.289
CADD_phred	GERP++_RS	phyloP46way_placental	phyloP100way_vertebrate	SiPhy_29way_logOdds			
22.4	5.57	2.78	9.242	19.517

## Discussion

As mentioned above, juvenile GM1 gangliosidosis is inherited in an autosomal recessive pattern and patients with this form experience neurodevelopmental regression. Clinical symptoms of GM1 gangliosidosis type I are presented during the first month of life. However, in GM1 gangliosidosis type II patients have normal neurodevelopmental milestones until late infantile period (late infantile form) or late childhood period (juvenile form). Therefore, if molecular diagnosis is performed early in the pre-symptomatic cases with positive family history, effective approaches such as enzyme replacement therapy, cell therapy and bone marrow transplantation can be provided for those types of the disease with later onset. Currently, there is no simple biochemical test available for carrier screening in high risk individuals and families [19]. Enzyme activity level is previously reported to be less severely affected in Type II patients [18] and was reported normal in one of our patients. Vacuolated lymphocytes in peripheral blood smears which is a simple procedure in the diagnosis of metabolic disorders, were observed in all our patients; however, this finding is nonspecific and might not be reliable in the early course of disease. With increasing availability and reducing costs of genetic tests such as high throughput next generation sequencing, it might be feasible to screen pathogenic mutations of *GLB1* gene, at least for all newborns from families at high risk of the disease.

Up to now, more than 165 disease-causing mutations have been reported in various studies and some researchers have identified mutations with high frequency. For instance, Georgiou T. et al. described high prevalence of a founder mutation (Arg482His) in an isolated small village in Cyprus [20]. These data show that molecular diagnosis of GM1 gangliosidosis may be difficult with these huge number of reported mutations. Therefore, in our study, we performed unbiased high throughput next generation sequencing to identify pathogenic mutations in three patients with early childhood ataxia and neurodevelopmental regression. These patients were born of consanguineous marriage from same “Lurs” ethnic background in the southwest of Iran. The Lur or Lor are an Iranian people living mainly in southwest and south Iran. The territories occupied by Lurs include three provinces which include Luristan (the land of Lors), Bakhtiari and Kuh-Gilu-Boir Ahmed. The territories presently occupied by the Lurs have been inhabited by man for some 40,000 years. There are some Y chromosome haplotypes in Lurs with a frequency of more than 10%, much higher than the general population of Iran. Therefore, we speculate that the identified mutations found in our unrelated families (two from same city and one from another distant city) might be due to a founder effect in Lurs population. Although enough number of whole exome sequencing (WES) data from Lurs are not available to calculate F inbreeding coefficient, it can be mentioned that there are many common variations among these three families in this study, raising possibility of having a common ancestor. By obtaining family history and extending it to other affected individuals with remote family relations or to the people residing in the same village, we identified the fourth patient and also reports about many other affected individuals who passed away with similar clinical phenotypes. Sanger sequencing confirmed this mutation in our patients, their parents and extended family members. The identified mutation in *GLB1* gene seems to have complete correlation with GM1 gangliosidosis type II and 100% penetrance in homozygous individuals. Although heterozygous carriers for this mutation are not apparently sick, they are at risk of passing this deleterious mutation to their offsprings. Therefore, *GLB1* genetic test should be requested for individuals with childhood ataxia and family members of known patients who intend to have consanguineous marriage. Presence of consanguineous marriage, history of death with similar presentations in the family, progressive ataxia and neurodevelopmental regression help physicians to narrow down the lists of differential diagnosis and to recommend available genetic tests.

## Conclusions

A missense mutation in *GLB1* gene, which segregated with the disease phenotype as autosomal recessive form of juvenile GM1 gangliosidosis type II was identified in four patients from the southwest of Iran. The mutation is a rare previously reported pathologic mutation, which has not been previously documented in Iranian population. Our results confirm a link between *GLB1* gene mutation, ataxia and neurodegeneration in patients with juvenile gangliosiodis type II in Iran.

## Abbreviations

EEG: Electroencephalography
ERG: Electroretinography
GLB1: Galactosidase, beta 1
GM1: Gangliosidosis
LSDs: Lysosomal storage diseases
MAF: Minor allele frequency
MRI: Magnetic resonance imaging
VEP: Visual evoked potential
WES: Whole exome sequencing
